# Modified Altemeier Procedure as Management for Incarcerated Rectal Prolapse in a Young Healthy Male Patient: A Case Report and Literature Review

**DOI:** 10.3390/medicina60111872

**Published:** 2024-11-15

**Authors:** Leenah Abdulgader, Ebtesam Al-Najjar, Bayan Khasawneh, Abdullah Esmail

**Affiliations:** 1 Department of Surgery, University of Maryland Medical Center, Baltimore, MD 21201, USA; 2 Section of GI Oncology, Houston Methodist Neal Cancer Center, Houston Methodist Hospital, Houston, TX 77030, USA

**Keywords:** Altemeier procedure, incarcerated rectal prolapse, surgery and sigmoid colostomy

## Abstract

Rectal prolapse (RP) is a rare condition presenting as a partial or complete protrusion of the rectum or as mucosa through the anal canal, and it usually occurs in the elderly or females with multiple risk factors. An initial presentation of incarcerated RP is even rarer. We present a case of a previously healthy 39-year-old man who presented with an incarcerated RP that necessitated urgent perineal proctosigmoidectomy (Altemeier procedure), with diverting sigmoid colostomy, followed by a reversal of the colostomy three months later. This case highlights the importance of surgical management (the modified Altemeier procedure) for a patient with an incarcerated RP. There are no specific guidelines for management of RP; all the recommendations and latest approaches are patients-based approaches according to their presentations, risk factors, age, and gender.

## 1. Introduction

RP predominantly occurs in elderly patients, with an estimated prevalence of less than 0.5% of the general population [1,2]. It rarely affects young healthy patients with no risk factors, and it more rarely presents initially as an incarcerated or strangulated rectal prolapse in this group. It is thought to start initially as internal intussusception of the rectum, which can only be detected by defecography. At this stage, the internal prolapse does not pass through the anal canal; it can occur separately or progress to complete RP, or procidentia, which is a full-thickness protrusion of the rectum through the anal canal.

Both presentations can occur separately or with pre-existing pelvic floor disorders, such as cystocele, vaginal vault prolapse, and rectocele [3]. Several other factors are thought to be associated with RP, such as a redundant sigmoid colon, a deep pouch of Douglas, diastasis of the levator muscle, and loss of the vertical position of the rectum due to a weak rectosacral fascia. Most patients who have anatomical dysfunction complain of chronic straining and constipation. The prolapsed rectum stretches the internal and external anal sphincters, causing incontinence.

Fluoroscopic defecography helps to diagnose RP in the early stage before it progresses to complete prolapse and has a higher detection rate of pelvic floor anomalies rectocele, rectal prolapse, rectoanal intussusception, and perineal descent, allowing for imaging in a more natural position [4].

Dynamic magnetic resonance defecography provides information on both structural and functional abnormalities, allows for the evaluation of concomitant pelvic floor disorders, and demonstrates a clear pelvic anatomy. It is vital for patients who have undergone prior pelvic or perineal surgery [5]

Other important diagnostic modalities: anal manometry and endoanal ultrasonography to assess the anal sphincter function, including the resting and squeezing pressures, length of the functional anal canal, recto-anal inhibitory reflex activity during rectal distension, rectal sensation, rectal compliance, and defecation function, also mapping of the extent of sphincter injury [4]

Nonoperative management for patients who have internal RP or pelvic floor dysfunction includes defecation training, biofeedback therapy, which involves real-time training of pelvic muscle contraction and anal sphincter relaxation in coordination with rectal emptying, life style modification including dietary changes with 30–40 g of fiber daily, and performing aerobic exercise, in addition to use of stool softeners [6]. These treatments are improving the quality of life. However, surgery should be considered if conservative therapies fail after 2–3 months.

The surgical management of RP is widely discussed and divided into two main approaches, abdominal and perineal; each of these approaches has multiple procedures. The perineal approaches are usually for high-risk patients with multiple comorbidities, although it is associated with higher recurrence rate but less complication [7]. Many surgical options have been explored based on the age of the patient, operative risk, psychiatric illness, and prolapse complication [8].

The complication of complete RP includes damage of the rectum and surrounding structures, bleeding, ulceration, incarceration, strangulation, gangrene, perforation, and intra-abdominal sepsis that could lead to multi-organs failure and death.

The objective of this literature is to review the surgical management of RP and to determine the appropriate procedure for the right patient.

## 2. Case Presentation

**Figure 1:**
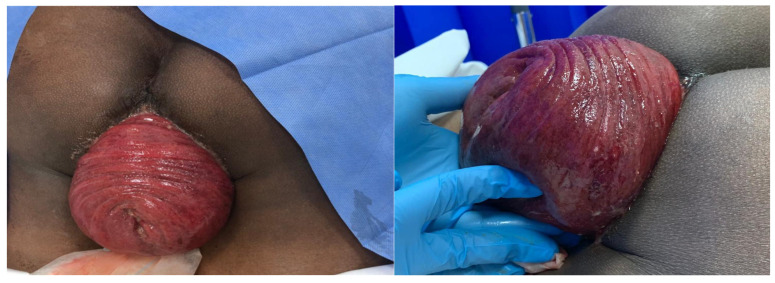
Complete incarcerated rectal prolapse.

**Figure 2:**
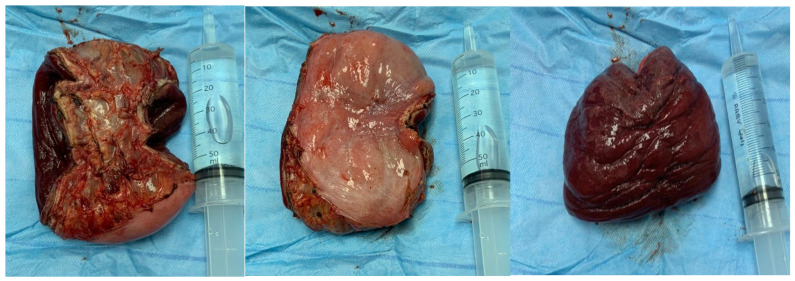
Resected specimen of the rectum and sigmoid colon.

A 39-year-old man, a smoker who had no medical or surgical history, was not on any medication, and worked as a school-bus driver, presented to the emergency department with a complaint of chronic constipation that was followed by a sudden large, painful, irreducible rectal mass protrusion through the anus after excessive straining. This was associated with mild abdominal pain, mucus leak, and minimal rectal bleeding. He had experienced no previous attacks and denied a history of hemorrhoids, any anorectal conditions, and homosexuality. The rest of the systemic review was unremarkable. Abdominal examination showed no signs of peritonitis or intestinal obstruction. A digital rectal exam in the prone position revealed a complete irreducible, congested prolapse of the rectal wall, with rectal concentric folds at the apex of the prolapse and multiple small areas of ulceration with no active bleeding. Manual reduction of the prolapsed part of the rectum was attempted, in addition to dextrose 50% wet gauze application under sedation, but it was unsuccessful (Figure 1). Thus, the patient was prepared for an emergency operation. A trial of perineal manual reduction under general anesthesia with a muscle relaxant was performed unsuccessfully. Trying to reduce the sigmoid colon through the lower midline laparotomy by performing a sigmoidectomy, which is the standard procedure for young patients with no risk factors for prolapse, was unsuccessful due to a severely congested full-prolapsed segment of the rectosigmoid; also, the Altemeier procedure alone carries a high risk of recurrence. Therefore, resection of the prolapsed rectosigmoid was performed using the perianal approach and reduction of the remnant rectum into the peritoneum cavity and fixed with a non-absorbable suture to facilitate the reversal of the colostomy (Figure 2); the remnant segment of the intra-abdominal sigmoid colon was resected, and an end-descending colostomy maturation as the distal end of the rectosigmoid junction was severely congested and at high risk of anastomosis disruption if we performed anastomosis as a single-step procedure. We preferred to give the tissue time for the edema to resolve before appropriate assessment of the pelvic floor and anal sphincter. The postoperative period was uneventful. Surgical pathology revealed a segment of the colon measures 10 cm in length and 20 cm in circumference with 1.5 cm wall thickness, and the mucosa shows irregular ulceration with lymphocyte infiltration with no atypia.

### Follow-Up

The patient was re-admitted three months later for colostomy reversal with a 31 mm end-to-end anastomosis using a circular stapler after thorough assessment. The entire colon and the rectum were evaluated by colonoscopy, which revealed no obvious gross pathologies, and the distal end of the rectum was healthy with no signs of mucosal atrophy. Also, anal manometry was performed and showed a normal anal tone. The patient tolerated the procedure well and was discharged in good condition after evidence of return of bowel function.

The surgical incision was well healed, with no signs of infection and no evidence of recurrence in the two weeks following the surgery. The patient was followed up in 3, 6, 9, and 12 months post-operation and showed no signs of prolapsed or anal continence for feces and gasses.

## 3. Discussion

The estimated prevalence of RP in the adult population is about 2.5/100,000, affecting women more than men [9]. About 2–4% present with a strangulated RP [10,11]. The exact pathophysiologic mechanism of RP is unclear. However, Zhai et al. proposed that the development of RP may be primarily attributed to electrophysiological and histological abnormalities, including factors such as weak pelvic floor support and denervation of the anal levator and sphincter muscles [12].

The prevailing theories are sliding herniation and progressive internal intussusception, resulting in protrusion through the anus. RP is usually seen in patients who have coexisting pathological and anatomical risk factors and prolonged symptoms prior to presentation. When the reduction of an incarcerated rectum fails, techniques such as sedation and the application of a hypertonic solution can help reduce it before surgical intervention. If these fail or in the case of rectum necrosis, surgical intervention is mandatory to restore the anatomical position of the digestive tract and enhance function. In emergency settings, only rectosigmoid resection using a perineal approach or the Altemeier procedure, with or without a diverting stoma, can be proposed [13].

The historical literature describes various surgical techniques and procedures for RP, which can be classified into an abdominal technique; posterior suture rectopexy with or without sigmoid resection, Ripstein mesh rectopexy, Ivalon sponge repair (Wells procedures), Ventral mesh rectopexy (Orr-Loygue procedure); and a perineal approach (i.e., Thiersch Altemeier, Delorme) [14]. Each of these surgical techniques presents distinct advantages and disadvantages. The perineal approaches can be carried out under regional or spinal anesthesia, are less at risk for surgery-related morbidity, and involve less postoperative pain, with a higher recurrence rate. In contrast, the abdominal approaches require general anesthesia, are more at risk for surgery-related morbidity, and involve more postoperative pain, with the lowest recurrence rate. Therefore, we should be considerate when selecting the appropriate treatment for patients [15]. As one size does not fit all patients.

Thiersch procedure is to encircle the wire around the anus in the perianal space through a small incision one-inch posterior to the anus. It is rarely used these days due to high risk of recurrence [16]

Delorme’s procedure involves resection of the mucosa and plication of the rectal wall muscle. It is suitable for patients who have a history of prolapse repairs, previous pelvic surgery, or pelvic radiotherapy, or with a short segment of prolapse [17]. A retrospective review by Tanabe demonstrated that patients with a prolapse length of ≥3 cm had a significantly higher risk of recurrence after Delorme’s procedure [18].

Over 130 therapeutic options for RP have been described in the literature [3]. However, the Altemeier procedure (perineal rectosigmoidectomy with coloanal anastomosis) is one of the most common procedures used in emergency settings and is considered a safe procedure compared to the abdominal approach, especially in patients with comorbidities. Regarding the postoperative risk of mortality, the Altemeier procedure was shown to result in an incidence of 1.6% and an estimated risk of complication of around 3%, as well as a low recurrence rate compared to other procedures [19].

A case series by Purnamaet al. recommended strongly considering the Altemeier procedure for irreducible prolapse, regardless of the presence of necrosis [14]. This case report emphasizes the importance of urgent surgical intervention using the Altemeier procedure for patients with an incarcerated RP.

Posterior suture rectopexy with or without sigmoid resection, Ripstein mesh rectopexy, and Ivalon sponge repair (Wells procedures) are involved in the mobilization of the rectum and sigmoid colon with a difference in fixation of the rectum. In posterior suture rectopexy, a permanent suture is used to fix the lateral stalk of the rectum to the presacral fascia, with sigmoid resection for patients who have had constipation. The Ripstein procedure involved placing a strip of mesh anterior to the rectum and fixing it to the sacrum, then covering it with the peritoneum layer to prevent small bowel adhesion to the mesh. Wells procedure involves placing a sheet of Ivalon between the sacrum and posterior wall of the rectum, then folding it around to cover the ¾ of the rectal circumference [20].

Ventral mesh rectopexy is performed by placing a synthetic mesh over the anterolateral of the rectal wall, then fixing it to the sacral promontory after incising the peritoneum at the level of rectosigmid junction and extended inferiorly and then across the peritoneal reflection. This procedure can be conducted laparoscopically or robotically [20].

The American College of Surgeons National Surgical Quality Improvement Program (NSQIP) classifies RP complications into major and minor categories. Major complications include organ space infections, thromboembolism, cardioembolic, ventilator dependence, renal failure, and sepsis. Minor complications include surgical site infections and urinary tract infections. [14]. In addition, they found that a perineal approach was independently associated with a lower 30-day major and minor complication rate compared to abdominal procedures. [21]. These findings align with the results of a retrospective study by Trompetto et al. which involved 43 female patients with complete RP who underwent the Altemeier procedure. The study reported a major complication rate of 2.3% (one patient), with complications unrelated to ASA score, BMI, or age, and no 30-day mortality [21]. In contrast, the most recent study by Zhai et al. involved a case series of 12 patients with impaction RP, treated with a modified Altemeier procedure as an emergency intervention after conservative treatments failed; the study found that this modified procedure effectively reduced mortality, recurrence, and complication rates, highlighting its advantages over other approaches [12]. This enhances our case using the perineal approach, as the patient reported no complications at follow-up and experienced complete resolution of symptoms with no recurrence, significantly improving their quality of life.

Based on the 2024 literature review by Koimtzis et al., the study aimed to compare the two most commonly used intra-abdominal procedures for treating RP: resection rectopexy and mesh rectopexy. Koimtzis et al. found no statistically significant differences between the two methods in terms of operating time, length of stay, overall complication rate, surgical site infection rate, cardiopulmonary complications, improvement in constipation and incontinence, or recurrence rates. They concluded that both procedures offer similar short- and long-term outcomes. Thus, the choice of procedure should be individualized, considering the surgeon’s preference and expertise [22].

The management of rectal prolapse is complex. Selecting the appropriate surgical intervention requires careful consideration of factors such as the patient’s medical history, clinical symptoms, surgeon’s experience, and available hospital equipment. In cases of irreducible or incarcerated rectal prolapse, surgical options are particularly limited. When the abdominal approach is impractical or unfeasible, the perineal approach often becomes the necessary choice.

## 4. Conclusions

Our case report describes a rare instance of incarcerated RP in a young adult. The patient’s successful treatment with perineal proctosigmoidectomy underscores the effectiveness of the Altemeier procedure in managing this emergency condition. The follow-up confirmed that the patient’s anal tone was normal, and the distal end of the rectum was in excellent condition, with no evidence of mucosal atrophy.

## Data Availability

The data of this study that supports our results are available on request from the corresponding author.
